# Specific Loss of Histone H3 Lysine 9 Trimethylation and HP1γ/Cohesin Binding at D4Z4 Repeats Is Associated with Facioscapulohumeral Dystrophy (FSHD)

**DOI:** 10.1371/journal.pgen.1000559

**Published:** 2009-07-10

**Authors:** Weihua Zeng, Jessica C. de Greef, Yen-Yun Chen, Richard Chien, Xiangduo Kong, Heather C. Gregson, Sara T. Winokur, April Pyle, Keith D. Robertson, John A. Schmiesing, Virginia E. Kimonis, Judit Balog, Rune R. Frants, Alexander R. Ball, Leslie F. Lock, Peter J. Donovan, Silvère M. van der Maarel, Kyoko Yokomori

**Affiliations:** 1 Department of Biological Chemistry, School of Medicine, University of California, Irvine, California, United States of America; 2 Leiden University Medical Center, Center for Human and Clinical Genetics, Leiden, The Netherlands; 3 Institute for Stem Cell Biology and Medicine, Department of Microbiology, Immunology, and Molecular Genetics, David Geffen School of Medicine, University of California, Los Angeles, California, United States of America; 4 Department of Biochemistry and Molecular Biology, University of Florida, Gainesville, Florida, United States of America; 5 Division of Medical Genetics and Metabolism, Department of Pediatrics, University of California Irvine Medical Center, Orange, California, United States of America; University of Cambridge, United Kingdom

## Abstract

Facioscapulohumeral dystrophy (FSHD) is an autosomal dominant muscular dystrophy in which no mutation of pathogenic gene(s) has been identified. Instead, the disease is, in most cases, genetically linked to a contraction in the number of 3.3 kb D4Z4 repeats on chromosome 4q. How contraction of the 4qter D4Z4 repeats causes muscular dystrophy is not understood. In addition, a smaller group of FSHD cases are not associated with D4Z4 repeat contraction (termed “phenotypic” FSHD), and their etiology remains undefined. We carried out chromatin immunoprecipitation analysis using D4Z4–specific PCR primers to examine the D4Z4 chromatin structure in normal and patient cells as well as in small interfering RNA (siRNA)–treated cells. We found that SUV39H1–mediated H3K9 trimethylation at D4Z4 seen in normal cells is lost in FSHD. Furthermore, the loss of this histone modification occurs not only at the contracted 4q D4Z4 allele, but also at the genetically intact D4Z4 alleles on both chromosomes 4q and 10q, providing the first evidence that the genetic change (contraction) of one 4qD4Z4 allele spreads its effect to other genomic regions. Importantly, this epigenetic change was also observed in the phenotypic FSHD cases with no D4Z4 contraction, but not in other types of muscular dystrophies tested. We found that HP1γ and cohesin are co-recruited to D4Z4 in an H3K9me3–dependent and cell type–specific manner, which is disrupted in FSHD. The results indicate that cohesin plays an active role in HP1 recruitment and is involved in cell type–specific D4Z4 chromatin regulation. Taken together, we identified the loss of both histone H3K9 trimethylation and HP1γ/cohesin binding at D4Z4 to be a faithful marker for the FSHD phenotype. Based on these results, we propose a new model in which the epigenetic change initiated at 4q D4Z4 spreads its effect to other genomic regions, which compromises muscle-specific gene regulation leading to FSHD pathogenesis.

## Author Summary

Most cases of facioscapulohumeral muscular dystrophy (FSHD) are associated with a decrease in the number of D4Z4 repeat sequences on chromosome 4q. How this leads to the disease remains unclear. Furthermore, D4Z4 shortening is not seen in a small number of FSHD cases, and the etiology is unknown. In the cell, the DNA, which encodes genetic information, is wrapped around abundant nuclear proteins called histones to form a “beads on a string”–like structure termed chromatin. It became apparent that these histones are modified to regulate both maintenance and expression of genetic information. In the current study, we characterized the chromatin structure of the D4Z4 region in normal and FSHD patient cells. We discovered that one particular histone modification (trimethylation of histone H3 at lysine 9) in the D4Z4 repeat region is specifically lost in FSHD. We identified the enzyme responsible for this modification and the specific factors whose binding to D4Z4 is dependent on this modification. Importantly, these chromatin changes were observed in both types of FSHD, but not in other muscular dystrophies. Thus, this chromatin abnormality at D4Z4 unifies the two types of FSHD, which not only serves as a novel diagnostic marker, but also provides new insight into the role of chromatin in FSHD pathogenesis.

## Introduction

FSHD is the third most common heritable muscular dystrophy [1]. It is characterized by progressive weakness and atrophy of facial, shoulder, and upper arm musculature, which can spread to the abdominal and foot-extensor muscles [2]. It can be accompanied by hearing loss and retinovasculopathy. The genetics underlying FSHD are highly unusual, as no pathogenic mutation(s) of a disease causing gene(s) has been identified. Instead, the majority (>95%) of FSHD cases involve mono-allelic deletion of D4Z4 repeat sequences at the subtelomeric region of chromosome 4q (termed “4q-linked” FSHD, FSHD1A (OMIM 158900); designated as “4qF” in this study) [2]. There are between one and ten repeats in the contracted 4qter allele in FSHD patient cells, in contrast to up to 11∼150 copies in normal cells. In addition, <5% of FSHD cases are not associated with D4Z4 repeat contraction (termed “phenotypic” FSHD, FSHD2; referred to as “PF” in this study), and their etiology remains undefined.

How contraction of the 4qter D4Z4 repeats causes muscular dystrophy is not understood. A previous study reported the YY1-nucleolin-HMGB2 repressor complex binding to D4Z4, and it was postulated that reduction of the repeat number may result in decreased repressor complex binding, leading to derepression of neighboring genes [3]. Consistent with this model, overexpression of the neighboring 4q35 genes was demonstrated in the same study, and the same group recently showed that muscle-specific overexpression of the neighboring gene *FRG1* indeed causes muscular dystrophy in mice [4]. Curiously, however, microarray and quantitative expression studies by other laboratories revealed that many genes located elsewhere in the genome important for myoblast differentiation are dysregulated, but unanimously provided no evidence for abnormal upregulation of FRG1 and other 4q35 genes in FSHD [5]–[7]. Furthermore, the model cannot explain the mechanism of phenotypic FSHD in which there is no D4Z4 repeat contraction.

Cytological analyses revealed that the 4q telomeric region uniquely associates with the nuclear periphery, consistent with the hypothesis that this region is heterochromatic [8],[9]. However, since the D4Z4 repeat contraction in 4qF did not lead to any significant localization changes, the functional relevance to FSHD remains uncertain [8],[9].

A recent study demonstrated that the 4qter D4Z4 region is hypermethylated at the DNA level in normal cells, but is hypomethylated in both 4q-linked and phenotypic FSHD [10]. This was the first evidence that 4qter D4Z4 is also involved in phenotypic FSHD. DNA methylation is an important mechanism for epigenetic regulation of gene transcription, and is generally associated with transcriptional silencing [11]. Thus, the results suggested that the D4Z4 repeat array organizes a transcriptionally suppressive heterochromatic environment, which is disrupted in FSHD. However, DNA hypomethylation, more severe than that seen in FSHD, at D4Z4 was also observed in another hereditary disorder, the “immunodeficiency, centromere instability and facial anomalies (ICF)” syndrome, due to a mutation in DNA methyltransferase 3B (DNMT3B) [10],[12]. Since the clinical presentation of ICF syndrome shares no similarity with the FSHD disease phenotype [13], the relevance of DNA methylation changes in FSHD is unclear and the molecular events underlying the D4Z4-linked disease process remain an open question.

Here we report the characterization of the chromatin of the 4q and 10q D4Z4 repeats and a comparison between normal, FSHD and other muscular dystrophy cells. Our results demonstrate that there is a distinct change of histone modification and downstream factor binding that is specifically associated with both 4q-linked and phenotypic FSHD, suggesting that epigenetic alteration plays a critical role in FSHD pathogenesis.

## Results

**Figure 1:**
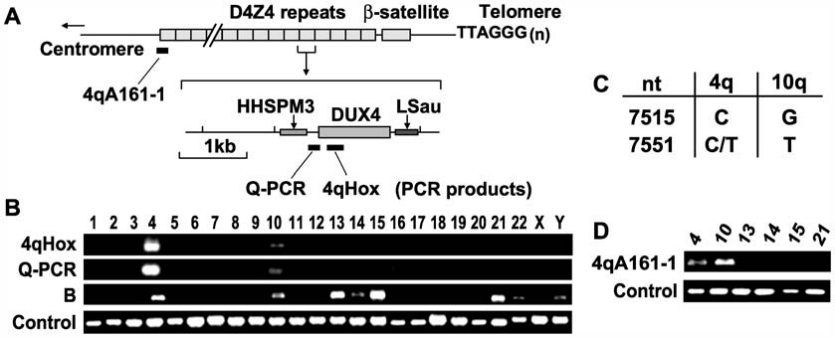
Specific PCR amplification of D4Z4 repeat sequences. (A) A schematic diagram of the 4qter D4Z4 repeat region and a single D4Z4 repeat. PCR products for Q-PCR and 4qHox primer pairs are indicated by black bars. The DUX4 ORF and a GC-rich sequence homologous to the low-copy repeat HHSPM3 [68] are shown. (B) PCR analysis of a DNA mapping panel consisting of genomic DNA isolated from mouse and hamster somatic cell hybrids containing individual human chromosomes using the 4qHox and Q–PCR primer pairs. The “B” PCR primer pair also binds to a region within D4Z4. However, it amplified not only chromosomes 4 and 10, but also several other chromosomes presumably due to crossreactivity to other D4Z4-like repeat sequences, and therefore, was not used for the experiments. For control PCR reactions, primers corresponding to the mouse β-globin locus [69] were used for the chromosome 1, 16, 17, 20, and 21 hybrids, while primers for hamster rDNA regions were utilized for the other hybrids. PCR analysis of additional mouse somatic cell hybrids for human chromosomes 4, 10, 13, 14, 15, and 21 also yielded similar results (data not shown). (C) Sequence polymorphisms between 4q and 10q D4Z4 [14],[70]. The nucleotide positions (nt) of the sequence polymorphisms are based on AF117653 in the GenBank/EMBL Nucleotide Sequence Database. (D) PCR analysis using the 4qA161-1 primer pair against genomic DNA from mouse somatic cell hybrids containing human chromosomes 4, 10, 13, 14, 15, and 21.

We examined the histone modification status of the D4Z4 region and whether this is altered in FSHD using chromatin immunoprecipitation (ChIP). Analysis of 4q D4Z4 chromatin has been difficult since D4Z4 repeat sequences are present in a similarly large cluster on both chromosomes 4q and 10q (though FSHD is associated only with D4Z4 contraction at 4q) [14]. In addition, D4Z4-like repeats are present on several other chromosomes [9]. We identified and used primer pairs that amplified products exclusively from chromosomes 4 and 10, but not from any other chromosome (Figure 1A). This was confirmed using DNA from somatic cell hybrids carrying individual human chromosomes as templates (Figure 1B and 1D). Furthermore, the regions amplified by the “Q-PCR” primer pairs contain specific nucleotide polymorphisms that allow us to distinguish 4q- and 10q-derived D4Z4 sequences (Figure 1C) [14]. Thus, in this study, the ChIP DNA amplified by Q-PCR primer pairs was cloned and sequenced to identify the chromosome of origin (Table S1).

### D4Z4 contains both heterochromatic and euchromatic domains

**Figure 2:**
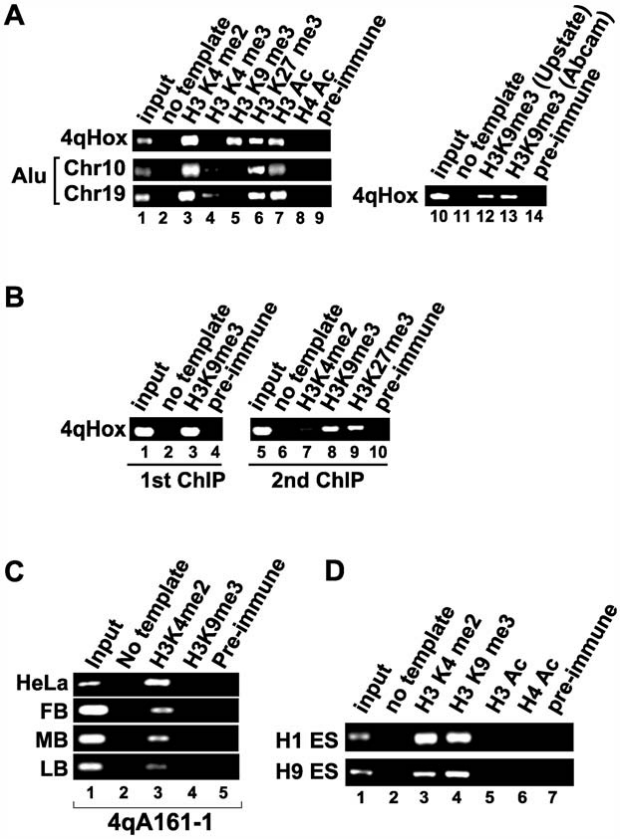
D4Z4 chromatin contains both euchromatic and heterochromatic histone modifications. (A) Antibodies specific for H3K4me2, H3K4me3, H3K9me3, H3K27me3, H3Ac, and acetylated H4 (H4Ac), as well as control preimmune IgG, were used for ChIP in HeLa cells. The ChIP DNA was amplified using 4qHox primers and primers specific for regions on chromosomes 10 and 19 containing short Alu repeat sequences. The presence of H3K9me3 was confirmed by two different antibodies (lanes 10–14) [18]. (B) Double-ChIP analysis of D4Z4 histone modifications. H3K9me3 ChIP (1st ChIP) was eluted and followed by the second (2nd) ChIP reactions using antibodies specific for H3K4me2, H3K9me3, H3K27me3, or preimmune IgG. The ChIP DNA was amplified using 4qHox primers. (C) The proximal region of the D4Z4 cluster is euchromatic. ChIP analysis of the first proximal D4Z4 repeat using the 4qA161-1 primer pair (See Figure 1D for sequence amplification specificity) was performed in HeLa, normal human fibroblasts (FB), myoblasts (MB), and lymphoblasts (LB). (D) Histone ChIP in human ES cells. ChIP DNA derived from the ES cell lines H1 and H9 was amplified by 4qHox primers. Antibodies used for ChIP are indicated at the top.

We found trimethylation of H3K9 (H3K9me3) and H3K27 (H3K27me3) at D4Z4, both of which frequently represent transcriptionally repressive heterochromatin [15],[16], as well as H3K4 dimethylation (H3K4me2) and H3 acetylation (H3Ac), which mark transcriptionally permissive euchromatin [17] (Figure 2A). H3K9me3 signals were confirmed by two different antibodies specific for H3K9me3 which have slightly different binding preferences [18] (Figure 2A, lanes 10–14). Recent studies demonstrated that H3K9me3 can also be associated with transcriptionally active gene regions [18],[19]. However, no significant H3K4me3, which is coupled to transcription-associated H3K9me3 [18], was detected using the same primer pairs (Figure 2A, lane 4). Although it is possible that H3K4me3 may be present elsewhere in the D4Z4 repeat, it is at least not present within the promoter and 5′ regions of the putative open reading frame (ORF) for DUX4 where 4qHox and the Q-PCR primers bind (Figure 1A). Furthermore, double-ChIP analysis revealed that H3K9me3 coincides with H3K27me3, but not H3K4me2, suggesting that the D4Z4 repeat cluster contains a distinct heterochromatic domain marked by both H3K9me3 and H3K27me3 as well as a euchromatic domain containing H3K4me2 (Figure 2B). Notably, PCR amplification of the first proximal D4Z4 repeat revealed that this end is euchromatic, consistent with a previous report that the region proximal to the D4Z4 repeat is euchromatic [6] (Figure 1A and Figure 2C). Both H3K4me2 and H3K9me3 are present at D4Z4 in human embryonic stem (hES) cells, suggesting that D4Z4 chromatin domains are marked by these histone modifications early in development and are maintained during differentiation (Figure 2D). This is in contrast to H3Ac, which is absent in hES cells and appears to be added at later stages (compare Figure 2A, lane 7 and Figure 2D, lane 5). Taken together, unlike the previous model that implies that D4Z4 is a uniformly transcriptionally repressive domain [3], we found that D4Z4 repeats are composed of both euchromatic and heterochromatic domains with possibly the proximal repeats being euchromatic. Importantly, the presence of both 4q- and 10q-specific nucleotide polymorphisms (Figure 1C) was confirmed by sequencing of the ChIP DNA, indicating that a similar spectrum of histone modifications are present in the 4q and 10q D4Z4 regions (Table S1).

### H3K9me3 is specifically lost in both 4q-linked and phenotypic FSHD

**Figure 3:**
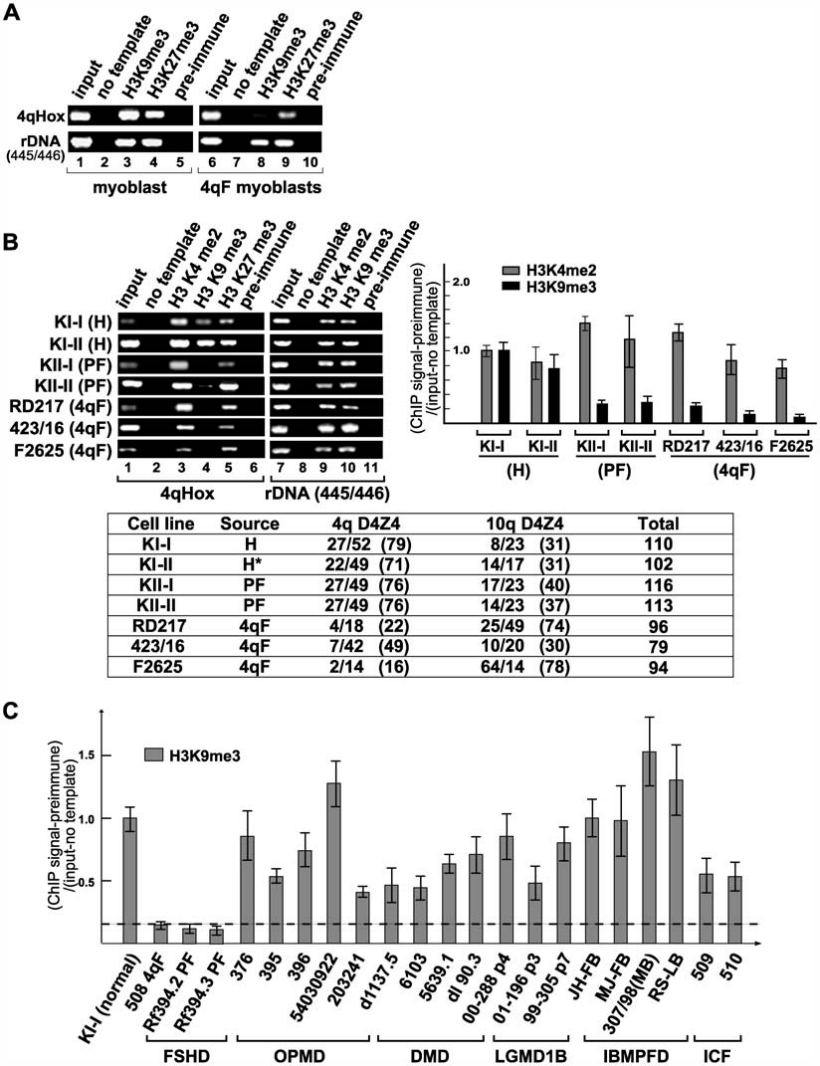
Histone H3 lysine 9 trimethylation is specifically lost at D4Z4 in both 4q-linked and phenotypic FSHD. (A) ChIP analysis of H3K9me3 and H3K27me3 at D4Z4 in normal and FSHD (4qF) myoblasts. The rDNA region (445/446) serves as a positive control. (B) H3K9me3 is specifically lost in FSHD fibroblasts. Endpoint PCR analysis with 4qHox primers by agarose gel electrophoresis and quantitation of real-time PCR with Q-PCR primers are shown. The rDNA region, which was positive for HP1 and cohesin binding, was used for comparison (445/446) (See Figure 5). PCR signals were normalized with preimmune, input, and no template PCR signals. Primary cells derived from healthy (normal) (H), phenotypic (PF), and 4q-linked (4qF) individuals were analyzed as indicated. D4Z4 repeat numbers for 4q and 10q alleles as well as the total D4Z4 repeat numbers are shown in the table. The asterisk indicates a clinically unaffected individual with DNA hypomethylation at D4Z4, whose two offspring developed phenotypic FSHD (KII-I and KII-II). (C) H3K9me3 ChIP analysis of different muscular dystrophy patient cells. The graph contains one 4qF (508) and two PF (Rf394.2 and Rf394.3) patient fibroblast samples, five OPMD patient fibroblast samples carrying alanine repeat insertions in the PABPN1 gene (376, 395, 396, 54030922, and 203241), four DMD patient fibroblast samples with mutations in the dystrophin gene (d1137.5, 6103, 5639.1, and dl90.3), three LGMD patient fibroblast samples with heterozygous mutations in the LMN gene (00–288, 01–196, 99–305) [55],[56], two ICF patient fibroblast samples [57], and four IBMPFD patient samples (two fibroblast and two lymphoblast) with mutations in the VCP gene (JH-FIB, MJ-FIB, 307/98, and RS-LCL) [58]. The KI-I (normal) fibroblast sample serves as a control. H3K9me3 was also retained in two additional control fibroblast samples (302, 557/96) (data not shown).

We next examined the chromatin modifications in FSHD patient-derived primary cells compared to normal cells from healthy individuals. The H3K9me3 signal at D4Z4 was significantly decreased in D4Z4-contracted FSHD myoblasts and fibroblasts while H3K27me3 and H3K4me2 remained unaffected (Figure 3A and 3B, 4qF). Importantly, the loss of H3K9me3 is site-specific because no significant change was observed at the ribosomal DNA (rDNA) region (Figure 3A, lower panels, and Figure 3B, lanes 7–11) or in the amount of total H3K9me3 detected by western blot (data not shown). Similarly, no loss of H3K9me3 was observed at other repeat sequences, including chromosome 1 α-satellite and satellite 2, chromosome 4 α-satellite, NBL2, DXZ4, and RS447, in FSHD patient cells compared to normal cells (Figure S1). The failure to detect H3K9me3 at D4Z4 is not due to an insufficient number of D4Z4 copies since the ChIP signals were normalized to input DNA to reflect D4Z4 repeat number changes, and the loss of H3K9me3 was also observed in phenotypic FSHD (PF) cells with no repeat contraction. It is unlikely to be the result of a drastic change in antibody accessibility since H3K27me3, which resides in the same region according to the double-ChIP results (Figure 2B), is unchanged (Figure 3A and 3B). The persistence of H3K27me3 at D4Z4 also eliminates the possibility that only one allele is intrinsically organized as heterochromatin and deletion of this particular allele leads to FSHD. This is in agreement with previous observations that there is no clear paternal or maternal bias of disease transmission suggestive of imprinting, which could differentially organize the chromatin structure of the two alleles [2],[20]. Consistent with this, no significant difference in subnuclear localization of the two 4qter regions was found by the previous FISH analyses [8],[9].

Interestingly, the total numbers of D4Z4 repeat copies (i.e. the numbers of 4q and 10q repeats combined) are comparable between normal and FSHD patient cells (Figure 3B, bottom panel). Since the analysis in normal cells indicate that 10q D4Z4 also contains similar H3K9me3 modification (see above), the low level of H3K9me3 ChIP signal in FSHD patient cells cannot simply be attributed to the chromatin change at 4q D4Z4. This suggests that the loss of H3K9me3 also occurs at 10q D4Z4. This is further supported by the fact that both 4q and 10q polymorphisms were found in the residual H3K9me3 ChIP Q-PCR products of PF (KII-I) and 4qF (RD217) samples (Table S1). The results provide the first evidence that 10q D4Z4 chromatin is co-regulated with 4q D4Z4 chromatin and undergoes similar loss of H3K9me3 in FSHD.

**Figure 4:**
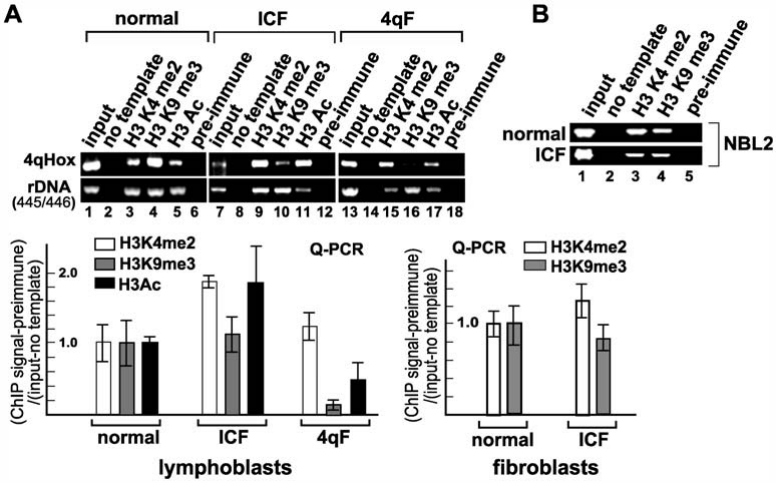
H3K9me3 at D4Z4 is maintained in ICF patient cells. (A) ChIP analysis by endpoint PCR using 4qHox primers was performed using normal, ICF, and 4q-linked FSHD (4qF) lymphoblasts with antibodies specific for H3K4me2, H3K9me3, and H3Ac, and preimmune IgG as indicated at the top. The rDNA region (445/446) serves as a positive control. ChIP analysis by real-time PCR using Q-PCR primers for H3K4me2, H3K9me3, and H3Ac is also shown. Similar results were obtained with ICF fibroblasts (Q-PCR results are shown at the bottom right). (B) H3K9me3 is intact at the NBL2 repeat region in ICF cells. Similar ChIP analysis was performed using PCR primers specific for the NBL2 repeat sequence.

The loss of H3K9me3 at D4Z4 was observed not only in FSHD patient myoblasts and fibroblasts, but also in lymphoblasts, indicating that this chromatin change is not a mere non-specific epiphenomenon associated with the dystrophic state of the muscle cell (Figure 3A and 3B and Figure 4A). Presently, we have examined 14 normal and 14 FSHD patient cell samples of different origins and obtained consistent results. Importantly, no significant loss of H3K9me3 at D4Z4 was observed in cells from Duchenne muscular dystrophy (DMD), limb-girdle muscular dystrophy (LGMD), oculopharyngeal muscular dystrophy (OPMD), and inclusion body myopathy associated with Paget's disease of bone and frontotemporal dementia (IBMPFD) (Figure 3C). Therefore, the loss of H3K9me3 at 4q and 10q D4Z4 appears to be a specific change uniquely associated with both 4q-linked (4qF) and phenotypic (PF) FSHD.

### Loss of H3K9me3 is distinct from DNA hypomethylation

**Figure 5:**
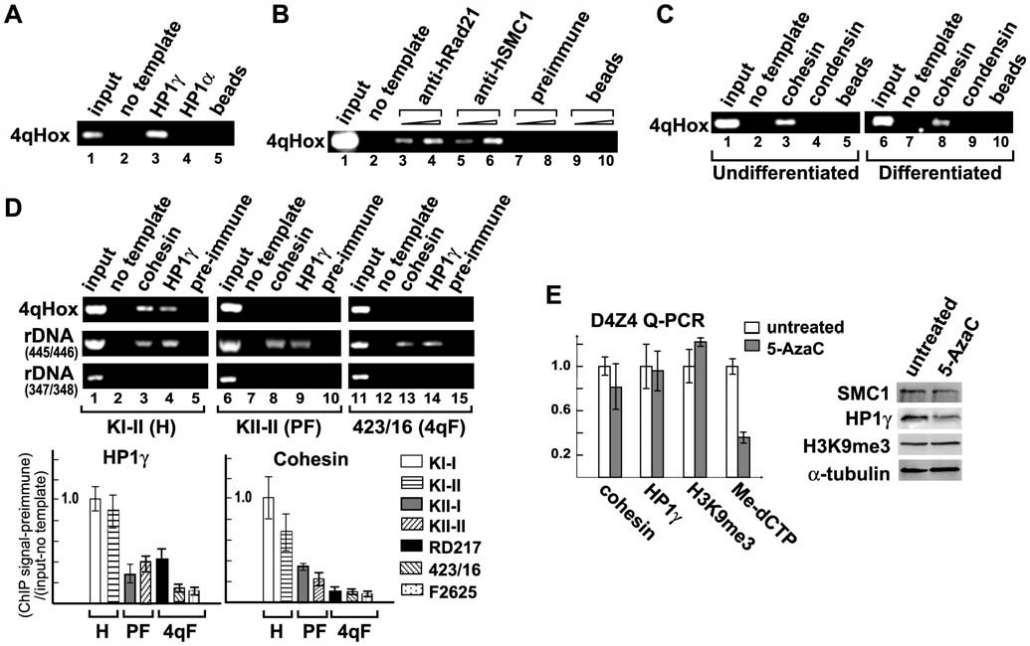
The binding of HP1γ and cohesin to D4Z4 is lost in FSHD patients. (A) HP1γ, but not HP1α, binds to D4Z4 in HeLa cells. 4qHox endpoint PCR of ChIP DNA using antibodies against HP1γ and HP1α is shown. Immunoprecipitation with protein A beads alone serves as a negative control. (B) Comparison of ChIP analyses using antibodies specific for two different subunits of cohesin (hSMC1 and hRad21). Preimmune IgG and protein A beads alone were used as negative controls. Two different amounts of ChIP DNA were used for endpoint PCR with 4qHox primers as indicated. The remainder of the cohesin ChIP experiments were carried out using anti-hRad21 antibody. (C) Cohesin binds to the 4qHox region in undifferentiated and differentiated primary human myoblasts. Cohesin ChIP was compared to that of condensin, another major SMC-containing complex, and protein A beads control. (D) ChIP PCR analyses using 4qHox primers of HP1γ and cohesin binding in H, PF and 4qF fibroblasts as in Figure 3B. Representative samples of the 4qHox PCR products on an agarose gel are shown. PCR primers corresponding to the rDNA locus serve as positive (445/446) and negative (347/348) controls for HP1 and cohesin binding. Real-time PCR analysis using Q-PCR primers of HP1γ and cohesin ChIP is shown underneath. A similar loss of HP1γ and cohesin was also observed in 4qF myoblasts (data not shown). (E) The effect of DNA hypomethylation on cohesin and HP1γ binding and H3K9me3. HeLa cells were treated with 5-AzaC and ChIP-PCR assays were performed using antibodies specific for Rad21 (“cohesin”), HP1γ and H3K9me3 and Q–PCR primers specific for D4Z4. Hypomethylation of DNA was confirmed by MeCIP using antibody specific for 5-methylcytidine. The ChIP and MeCIP signal intensity was normalized by genomic DNA input control and pre-immune control. No significant decrease of cohesin and HP1γ binding and H3K9me3 was observed. Western analysis of cohesin, HP1γ and H3K9me3 levels in untreated and 5-AzaC-treated cells is also shown.

DNA and heterochromatic histone methylation are often co-regulated [21]. Although DNA methylation is more frequently a downstream consequence of H3K9 methylation [22], DNA methylation in some instances was shown to promote H3K9me3 [23]. Thus, we next addressed whether the loss of H3K9me3 is simply a downstream event of DNA hypomethylation previously observed in FSHD and clinically unrelated ICF syndrome cells [10]. We found that H3K9me3 is largely intact at D4Z4 in ICF cells, though there appears to be an increase in H3K4me2 and H3Ac, as indicated by ChIP analysis (Figure 3C and Figure 4A). Similarly, H3K9me3 is unaffected at another non-satellite repeat sequence called NBL2 in ICF cells, which was also shown to be DNA-hypomethylated in these cells (Figure 4B) [12]. Furthermore, no significant loss of H3K9me3 was observed in cells from a clinically unaffected individual with significant DNA hypomethylation at D4Z4 (Figure 3B, KI-II) [10]. Finally, treatment of cells with 5-Azacytidine (5-AzaC), which blocks DNA methylation, did not affect H3K9me3 despite the significant reduction of DNA methylation at D4Z4 (Figure 5E). Taken together, DNA methylation is not required for H3K9me3 at D4Z4, and H3K9me3 loss clearly distinguishes FSHD from ICF, implying that loss of H3K9me3 at D4Z4, rather than DNA hypomethylation, is causally involved in FSHD.

### HP1γ and cohesin are specifically recruited to D4Z4, which is lost in FSHD

What happens as a result of the loss of H3K9me3 at D4Z4? To investigate the consequences of H3K9me3 loss in FSHD, we examined factors that bind to this region. Heterochromatin binding protein HP1 is recruited to heterochromatic regions by direct binding to the methylated H3K9 residue and plays an important role in transcriptional silencing [24],[25]. Swi6, an HP1 homolog in *S. pombe*, was also shown to recruit the essential sister chromatid cohesion complex “cohesin” to the pericentromeric heterochromatin where it mediates centromeric sister chromatid cohesion critical for mitosis [26],[27]. Although the study in yeast indicated that cohesin does not play any role in transcriptional repression at heterochromatic regions [27], HP1 and cohesin are valid candidates for the downstream effectors of H3K9me3 at D4Z4 in human cells. In mammals, there are three HP1 variants: HP1α, HP1β and HP1γ. We found that HP1γ specifically binds to D4Z4 (Figure 5A). Cohesin binding to D4Z4 was also detected using antibodies against two of its subunits (i.e., hSMC1 and hRad21), indicating the presence of the holo-complex (Figure 5B). Cohesin binding was observed in both undifferentiated myoblasts and differentiated (mitotically inactive) myotubes, suggesting a role beyond mitosis at this site (Figure 5C). Importantly, similar to H3K9me3, HP1γ and cohesin binding was also compromised at D4Z4, but not at the rDNA, DXZ4, and chromosome 1 α-satellite and satellite 2 repeat regions where H3K9me3 appears intact, in both 4qF and PF cells (Figure 5D and Figure S2). The results indicate that H3K9me3, HP1γ and cohesin form heterochromatin at D4Z4, and suggest that the loss of HP1γ and cohesin binding to D4Z4 is a significant downstream consequence of the loss of H3K9me3 in FSHD. Similar to H3K9me3, treatment of cells with 5-AzaC did not affect cohesin and HP1γ binding to D4Z4, further separating H3K9me3 and HP1γ/cohesin binding from DNA methylation (Figure 5E).

### SUV39H1 is responsible for H3K9me3, which is necessary but not sufficient for HP1γ/cohesin recruitment to D4Z4

**Figure 6:**
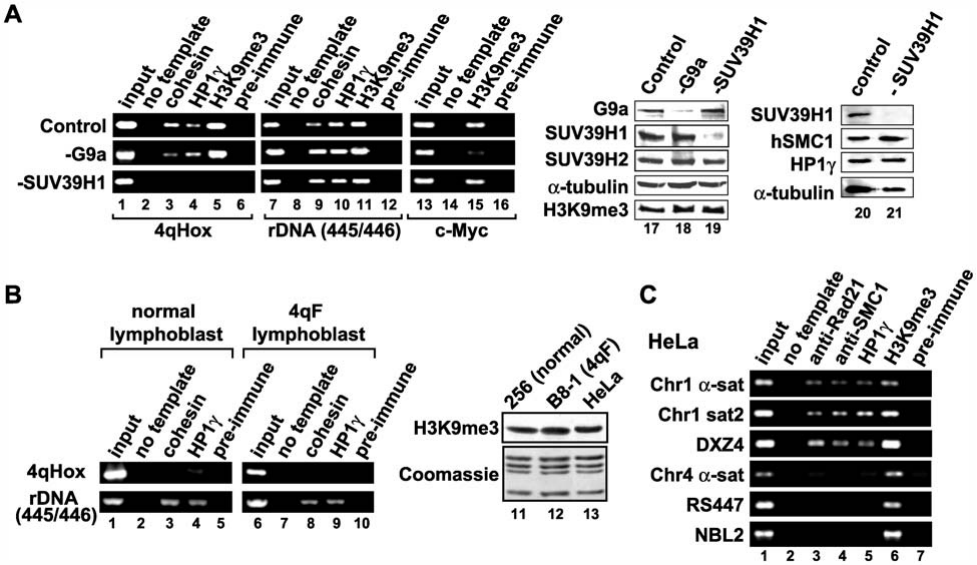
SUV39H1 HMTase is solely responsible for H3K9me3 at D4Z4, which is necessary, but not sufficient, for the recruitment of HP1γ and cohesin. (A) SUV39H1 is responsible for H3K9me3 and HP1γ/cohesin association at D4Z4. HeLa cells were treated with siRNA specific for SUV39H1, G9a, or control siRNA, and ChIP analysis using 4qHox primers was performed for the presence of cohesin, HP1γ and H3K9me3 (lanes 1–16). Preimmune IgG serves as a negative control. The rDNA (445/446) and c-Myc regions were used for comparison. Western-blot analysis of G9a and SUV39H1 siRNA depletion is also shown (lanes 17–21). Depleted proteins are indicated at the top and proteins detected by western blot analysis are indicated on the left. α-tubulin serves as a loading control. (B) HP1γ and cohesin binding to D4Z4 is cell type-specific. ChIP analysis of D4Z4 and rDNA regions was performed using normal and 4qF lymphoblasts (lanes 1–10). Western blot analysis comparing the level of H3K9me3 between HeLa and lymphoblasts (256 (normal) and B8-1 (4qF)) is also shown (lanes 11–13). Coomassie staining of core histones is included as a loading control. (C) Not all H3K9me3-positive repeats are bound by HP1γ and cohesin. Six different repeat sequences (as in Figure S1) were tested for cohesin and HP1γ binding in HeLa cells. While H3K9me3 was detected at all six repeat sequences tested, cohesin and HP1γ binding was found at only three repeats (α-sat and sat2 on chromosome 1 and DXZ4).

**Figure 7:**
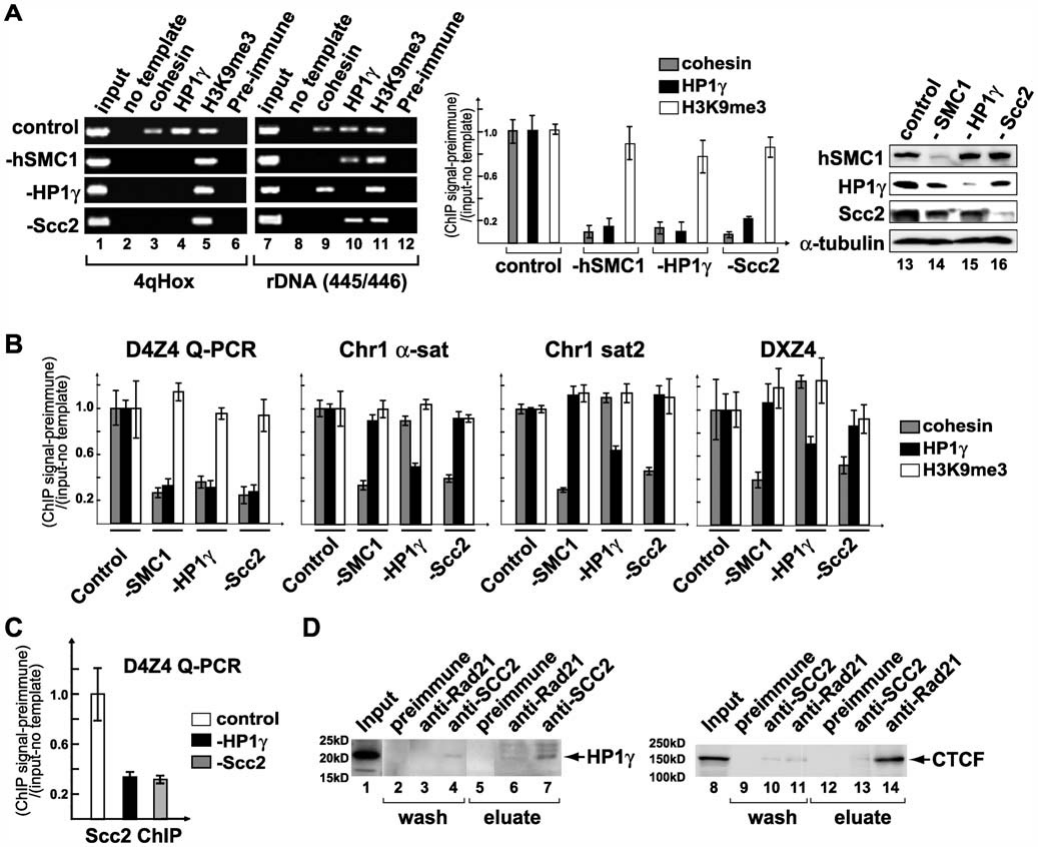
D4Z4-specific co-recruitment of HP1γ, cohesin, and cohesin loading factor Scc2. (A) Binding of HP1γ and cohesin to D4Z4 is interdependent. ChIP analysis of HeLa cells after individual depletion of the cohesin subunit hSMC1, HP1γ, or the cohesin loading factor Scc2 by siRNA as indicated (lanes 1–12). Cohesin and HP1γ binding was compared between D4Z4 and rDNA (445/446). Real-time PCR analysis using Q–PCR primers is shown underneath. Western blot analysis of hSMC1, HP1γ and Scc2 depletion is also shown (lanes 13–16). (B) HP1γ and cohesin binding do not affect each other at other repeat sequences. Realtime PCR analysis of Rad21 (“cohesin”), HP1γ and H3K9me3 ChIP DNA from HeLa cells treated with control, SMC1, HP1γ, or Scc2 siRNA as indicated using Q-PCR primers specific for D4Z4, α-sat and sat2 repeat sequences on chromosome 1, and DXZ4 (as in Figure S2). (C) Scc2 binding to D4Z4 is compromised by HP1γ depletion. Realtime PCR analysis of Scc2 ChIP DNA from HeLa cells treated with control, HP1γ, or Scc2 siRNA as indicated using Q-PCR primers specific for D4Z4. (D) Coimmunoprecipitation (co-IP)–western blot analysis of cohesin and Scc2 interaction with HP1γ. HeLa nuclear extracts were used for co-IP using antibody specific for Scc2 or cohesin (Rad21) as previously described [53],[61]. After low-salt washes, precipitated materials were eluted with 1.0 M KCl (“wash”) and further eluted with 2.0 M guanidine-HCl (“eluate”). Eluted proteins were analyzed by SDSPAGE and western blotting using antibody specific for HP1γ. For comparison, a similar co-IP analysis was performed and probed with antibody specific for CTCF.

The methyltransferase responsible for H3K9me3 and the relationship between H3K9me3, HP1γ and cohesin were addressed using small interfering RNAs (siRNAs). SiRNA against SUV39H1, which has no effect on SUV39H2, abolished H3K9me3 at D4Z4 but not at rDNA, suggesting that SUV39H1 has a non-redundant function at D4Z4 (Figure 6A). Supporting this notion, depletion of G9a, another H3K9 methyltransferase, decreased H3K9me3 at the c-Myc region [28], but had no effect at D4Z4 or rDNA (Figure 6A). Abolishment of H3K9me3 by SUV39H1 depletion also impaired HP1γ and cohesin binding at D4Z4 but not at rDNA, confirming that SUV39H1-mediated H3K9me3 is necessary for HP1γ and cohesin binding specifically at D4Z4. Neither HP1γ nor cohesin depletion affected the level of H3K9me3 at D4Z4, placing them downstream of H3K9me3 (see Figure 7A, lane 5).

Interestingly, HP1γ and cohesin binding to D4Z4 is significantly low in normal lymphoblasts, even with intact H3K9me3 at D4Z4 (Figure 4A, lane 4), when compared to other cell types (compare Figure 6B to Figure 5). This is not due to a general decrease of HP1γ and cohesin binding in lymphoblasts since HP1γ and cohesin binding was clearly observed at four other repeat sequences tested (i.e., rDNA, α-satellite and satellite 2 on chromosome 1, and DXZ4) in both normal and FSHD lymphoblasts, similar to myoblasts and fibroblasts (Figure 6B and Figure S2). Furthermore, the total level of H3K9me3 is comparable between HeLa and both normal and FSHD lymphoblasts (Figure 6B, lanes 11–13). The results indicate that H3K9me3 is not sufficient and suggest that an additional factor(s), which may be expressed in a cell type-specific manner, is required for HP1γ and cohesin binding to D4Z4. The requirement for an additional factor(s) is also supported by the observation that not all H3K9me3-positive repeat sequences are bound by HP1γ and cohesin, even in the same cell sample (Figure 6C).

### Cohesin plays an active role in HP1γ recruitment to D4Z4

Similar to the recruitment of cohesin to pericentromeric heterochromatin in *S. pombe* [26],[27], HP1 is required for cohesin binding at D4Z4 (Figure 7A). Interestingly, depletion of HP1γ alone abolished cohesin binding at D4Z4, indicating that HP1α and HP1β cannot compensate for this function of HP1γ at this site. In contrast, depletion of HP1γ had no effect on cohesin binding to the rDNA region, α-satellite and satellite 2 repeats on chromosome 1, and DXZ4, most likely due to functional redundancy with other HP1 variants (Figure 7A (lanes 7–12) and B). Consistent with this notion, HP1α binding was detected at the α-satellite repeat, but not at D4Z4 (Figure 5A, lane 4; data not shown). Thus, HP1γ is uniquely involved in heterochromatin formation at D4Z4.

We found that the cohesin loading factor Scc2 [29] also binds to D4Z4, which was significantly decreased by depletion of HP1γ to an extent similar to the decrease caused by depletion of Scc2 itself (Figure 7C). Consistent with this, we found an interaction between the endogenous HP1γ and Scc2 by in vivo coimmunoprecipitation (co-IP) (Figure 7D). Although weak, the interaction is specific and partially resistant to a 1 M salt wash (Figure 7D, “eluate”). We found that HP1γ mainly interacts with Scc2, rather than cohesin (Figure 7D). Although it was originally shown that HP1 interacts with cohesin in *S. pombe* [27], the interaction of Scc2 with HP1 variants was reported in human cells [30] and more recently in *S. pombe* [31]. Interestingly, CTCF, another factor recently shown to recruit cohesin to its binding sites [32]–[34], interacts preferentially with cohesin but not Scc2 (Figure 7D), suggesting distinct modes of cohesin recruitment by these factors.

In *S. pombe*, cohesin is downstream of HP1, and does not play any role in HP1 recruitment [27]. Interestingly, we found that depletion of hSMC1 impairs HP1γ binding to D4Z4 (Figure 7A). Similarly, depletion of Scc2 abolished D4Z4 binding of not only cohesin but also HP1γ. Thus, the results provide the first evidence for an active role of cohesin in heterochromatin organization. This appears to be context-dependent, since the rDNA region, α-satellite and satellite 2 repeats on chromosome 1, and the DXZ4 region showed no effect on HP1γ binding following depletion of hSMC1 or Scc2 (Figure 7A and 7B).

## Discussion

**Figure 8:**
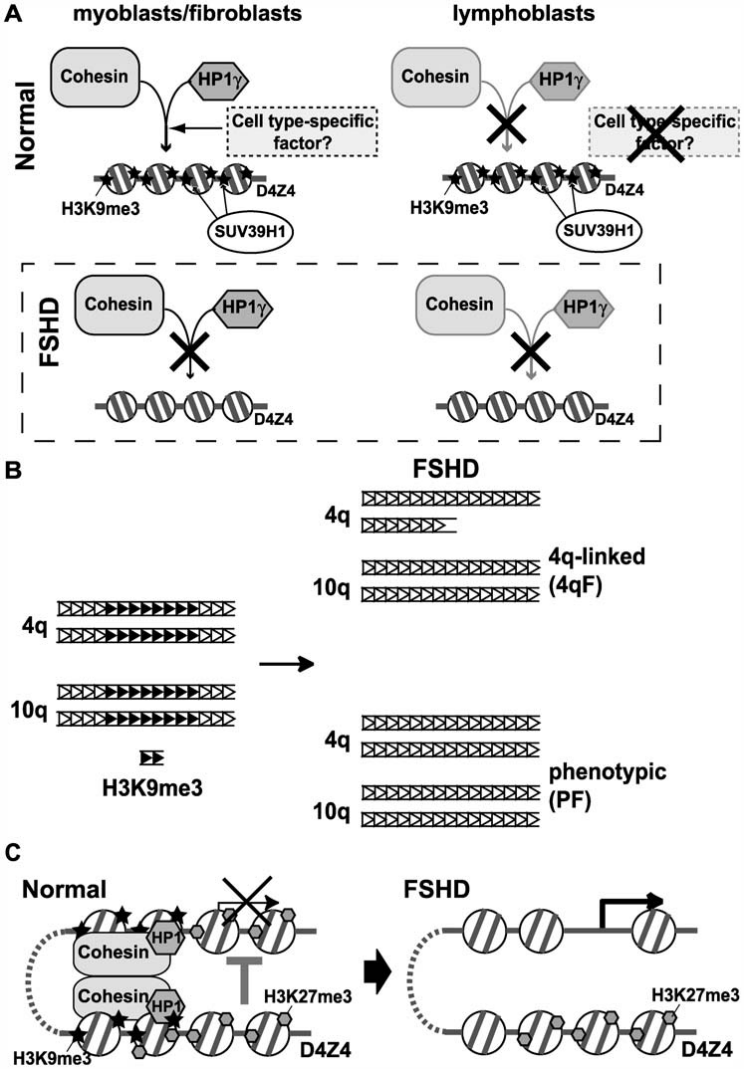
Schematic models of chromatin changes and the possible consequences in FSHD. (A) Schematic summary of the cell type-specific chromatin assembly at D4Z4 and its loss in FSHD. HP1γ and cohesin are co-recruited to D4Z4 that harbors SUV39H1-dependent H3K9me3 in certain cell types, including myoblasts and fibroblasts. In lymphoblasts, however, despite the presence of H3K9me3, HP1γ and cohesin fail to associate with D4Z4 raising the possibility that HP1γ and cohesin are involved in cell type-specific chromatin organization and that a putative cell type-specific factor(s) (or modification(s)) required for their recruitment may not be present in lymphoblasts. Thus, while loss of H3K9me3 at D4Z4 in FSHD has no consequence at D4Z4 in lymphoblasts, it leads to abolishment of HP1γ/cohesin binding in myoblasts, resulting in a detrimental effect on chromatin organization leading to muscular dystrophy. (B) Coordinated loss of H3K9me3 on 4q and 10q D4Z4 in 4qF and PF. H3K9me3 (shown by black triangles) clustered in the subdomains of D4Z4 repeat regions (distribution hypothetical) in normal cells is lost in both types of FSHD. (C) A possible model for the spreading of the epigenetic change at D4Z4 to other genomic regions in FSHD. HP1γ and cohesin may contribute to the physical interactions of the heterochromatic D4Z4 region with other genomic regions leading to the spreading of the silencing effect to putative target genes in normal cells. In FSHD, the loss of H3K9me3 (but not H3K27me3), HP1γ, and cohesin from D4Z4 results in loss of chromatin interaction and derepression of these genes leading to muscular dystrophy.

In this study, we found that the loss of histone H3K9me3 and its cell type-specific downstream effectors HP1γ and cohesin from D4Z4 repeats is the unifying molecular change in FSHD (Figure 8A). Importantly, this change was observed in both 4qF with D4Z4 contraction and PF without D4Z4 contraction. It was not found in ICF syndrome, despite its apparent similarity to FSHD with regard to D4Z4 DNA hypomethylation, or in other types of muscular dystrophies tested. This tight phenotype-epigenotype correlation strongly suggests that the loss of H3K9me3 at D4Z4 is critically involved in FSHD pathogenesis. Our results define a novel diagnostic marker for FSHD, and provide the first direct evidence for the specific changes of D4Z4 chromatin that are linked to FSHD.

### D4Z4 repeat clusters consist of euchromatic and heterochromatic domains, and only H3K9me3, but not H3K27me3, is lost from the heterochromatic domains in FSHD

Although D4Z4 was thought to be a uniformly transcriptionally repressive domain [3],[10], we found that D4Z4 regions contain a mixture of euchromatic and heterochromatic histone modifications; specifically, H3K4me2 and H3Ac as well as H3K9me3 and H3K27me3. These euchromatic and heterochromatic modifications are present in distinct domains within D4Z4 repeat clusters with the first proximal repeat being euchromatic (Figure 8B). Interestingly, only H3K9me3 is lost in FSHD, but not H3K27me3 from the heterochromatic region (Figure 8C). Thus, the chromatin change in FSHD is not a total loss of transcriptionally repressive heterochromatin. This is consistent with the fact that there apparently is no significant compensatory increase of euchromatic modifications, suggestive of expansion of euchromatic domains within D4Z4, in FSHD.

### Loss of H3K9me3 and D4Z4 contraction

PF and 4qF are genetically distinct. While the etiology of PF is unknown, our results revealed a correlation between the repeat contraction and the loss of H3K9me3 at D4Z4 in 4qF patient cells. This raises the possibility that repeat contraction leads to the loss of H3K9me3 at D4Z4 in 4qF. It is also formally possible that the upstream event that initially caused the repeat contraction might have also caused the loss of H3K9me3. It is less likely that the loss of H3K9me3 is the cause of repeat contraction, since there is no repeat number instability in phenotypic FSHD despite the similar loss of H3K9me3. Detection of H3K9me3 at D4Z4 in hES cells and multiple cell types indicates that H3K9me3 at this region is normally established early during development at a pluripotent stage, and is maintained throughout multi-lineage differentiation. The fact that H3K9me3 is lost even in lymphoblasts in FSHD patients indicates that this establishment process during early development may have gone awry.

Interestingly, our results indicate that contraction of one allele not only triggers the histone modification change (loss of H3K9me3) on the disease allele, but also affects H3K9me3 levels on other non-contracted 4q and 10q D4Z4 alleles, suggesting a functional communication between these homologous sequences perhaps reminiscent of transvection in *Drosophila* [35] (Figure 8B). This is in contrast to DNA hypomethylation, which appears to be restricted to the disease chromosome in FSHD [10],[13]. The dominant effect of contraction of one 4q D4Z4 allele on H3K9me3 at other D4Z4 alleles is consistent with the dominant nature of the disease and is in agreement with our results indicating that DNA hypomethylation is not required for the loss of H3K9me3. This strongly argues against the theory that only the contracted D4Z4 allele is involved in FSHD pathogenesis [3]. Rather, it is possible that both alleles of 4q D4Z4 as well as 10q D4Z4 may be involved in the disease process. Consistent with the coordinated chromatin changes observed, somatic pairing of 4q and 10q D4Z4 has been reported [36]. Although the mechanism is currently unclear, the results provide the first evidence that the initial genetic change (repeat contraction) spreads its effect to other genomic regions in 4qF. A similar coordinated loss of H3K9me3 at 4q and 10q D4Z4 was observed in PF, further emphasizing the significance of this phenomenon.

### Regulation of the SUV39H1 activity at D4Z4

We identified the histone methyltransferase (HMTase) SUV39H1, but not other HMTases, to be responsible for D4Z4 H3K9me3 (Figure 8A). This raises the possibility that misregulation of this enzyme activity is linked to the etiology of FSHD. However, no mutation in SUV39H1 itself (either at the promoter or gene region) in FSHD patient cells was found [13]. Consistent with this, the total level of H3K9me3 in the nucleus is similar between normal and FSHD cells. This suggests that a specific cofactor of SUV39H1, possibly important for its recruitment, and/or a specific histone demethylase acting at D4Z4, may be compromised in FSHD. It is plausible that PF results from a genetic mutation of such a factor. Further investigation of the site-specific SUV39H1 (or antagonizing histone demethylase) regulation will be important to understand FSHD's etiology and pathogenesis, and may shed new light onto the yet to be identified cause of PF. It is also interesting to note that there is a slight but consistent decrease in HP1γ binding to other repeat sequences tested in PF, but not 4qF, cells (Figure S2). Although the significance of this small decrease is currently unclear, this may reflect the distinct etiologies of PF and 4qF and may provide another clue to identify the genetic defect in PF.

### HP1γ and cohesin as cell type–specific downstream effectors

We established the loss of H3K9me3 at D4Z4 to be the signature change in both types of FSHD, but how does this epigenetic change lead to muscular dystrophy? We identified two major downstream effectors of H3K9me3, the heterochromatin binding protein HP1γ and cohesin, whose binding to D4Z4 is H3K9me3-dependent and, consequently, is severely compromised in FSHD. The data presented here argue for both factors having a role in FSHD pathogenesis. Importantly, while H3K9me3 at D4Z4 is seen in all cell types tested, the binding of HP1γ and cohesin to D4Z4 is cell type-specific, suggesting that their binding is involved in cell type-specific chromatin organization (Figure 8A). This restricted HP1γ/cohesin binding to D4Z4 may explain the tissue-specific FSHD disease phenotype, as their loss may be particularly deleterious to muscle function.

Interestingly, recent evidence suggests that cohesin is also involved in gene regulation. Although initially identified as a factor essential for mitosis, discoveries of mutations of cohesin components and the essential cohesin chromatin loading factor NIPBL/Scc2 in the developmental disorder Cornelia de Lange Syndrome (CdLS) strongly suggested the involvement of cohesin in developmental gene regulation [37]–[39]. The sequence-specific DNA binding transcription factor CTCF was found to recruit cohesin to many of its binding sites, where cohesin is involved in CTCF-dependent transcriptional regulation [32]–[34]. Accumulating evidence indicates that gene regulation can be affected by physical interaction between two distant chromosomal regions in *cis* and in *trans* in mammalian cells [40]–[43]. CTCF is known to be one such factor that exerts its transcriptional activity by directing long-distance chromatin interactions and loop formation, for example, in imprinting and X inactivation [44],[45]. Thus, the discovery that cohesin is an important mediator of CTCF transcriptional function raised the intriguing possibility that cohesin may dictate gene expression by facilitating such higher-order chromatin organization. Recent reports support this notion for cohesin at certain CTCF binding insulator sites [46],[47]. Similar to what was proposed for sister chromatid cohesion [48], cohesin may trap two distant chromatin fibers inside of its ring.

We failed to detect any significant binding of CTCF concomitant with cohesin at D4Z4 (data not shown), which is consistent with the fact that CTCF and heterochromatin are mutually exclusive [49]. However, cohesin may still function in a similar manner mediating long-distance chromatin interactions, together with HP1γ in the case of D4Z4 heterochromatin. In *Drosophila*, it was suggested that HP1 promotes interchromosomal association of heterochromatin, which may be important for coordinated gene silencing [50]. Evidence for gene silencing by association with distant heterochromatin was also found in mammalian cells, in which the temporal association of the terminal transferase (Dntt) gene with pericentromeric heterochromatin correlates with its silencing during thymocyte maturation in mice [51]. Thus, one possibility for the involvement of D4Z4 heterochromatin in gene regulation is that it makes contact with, and represses, distant target genes via long-distance chromatin: chromatin interactions by spreading a silencing effect in normal cells (Figure 8C). H3K27me3 found in the same region may also contribute to this by possibly recruiting the polycomb silencing complex. We hypothesize that in FSHD the loss of H3K9me3, and therefore of HP1γ and cohesin, results in the loss of this chromatin interaction, thereby causing abnormal derepression of these distant target genes that leads to the dystrophic phenotype (Figure 8C). There may be different sets of target genes for 4q and 10q D4Z4, both of which would be affected in FSHD due to the concomitant loss of H3K9me3. Interestingly, some evidence for change in local higher-order chromatin organization and nuclear matrix association in 4q-linked FSHD was recently reported [52]. However, this change appears to occur in the nearby regions outside of the D4Z4 cluster, and how D4Z4 contraction affects this is unclear. The same phenomenon has not been confirmed in phenotypic FSHD. In addition, since this change was shown to be restricted to the contracted allele and not other D4Z4 alleles, the relationship to the spreading of D4Z4 chromatin changes observed in the current study remains to be investigated. Further studies to examine the possible chromatin interactions and organization involving D4Z4 and their changes in FSHD may provide critical insight into the mechanism of FSHD pathogenesis.

## Methods

### Cells and DNA mapping panel

HeLa cells were grown as described previously [53]. The undifferentiated and differentiated normal myoblasts and the FSHD patient myoblasts were grown in SkBM-2 (Skeletal Muscle Cell Basal Medium, Cambrex Bio Science, NJ). Myoblast differentiation was induced by 2% horse serum as previously described [5]. Five normal and five 4q-linked FSHD myoblast lines were used. Control (KI-I, KI-II, NFGr), ICF (ICF1 and ICF2), 4q-linked FSHD (91RD217, 423/16, F2625, 508) and phenotypic FSHD (KII-I, KII-II, Rf394.2, RF394.3) fibroblasts were grown in DMEM/F-12 (1∶1) supplemented with 10% FBS, penicillin/streptomycin, 2 mM GlutaMAX-I (Invitrogen-Gibco, CA), 10 mM HEPES buffer and 1 mM sodium pyruvate [10],[54]. For comparison among different muscular dystrophies, one 4q-linked FSHD (508) and two phenotypic FSHD (Rf394.2 and Rf394.3) patient fibroblast samples, five OPMD patient fibroblast samples (376, 395, 396, 54030922, and 203241), four DMD patient fibroblast samples (d1137.5, 6103, 5639.1, and dl90.3), three LGMD patient fibroblast samples (00–288, 01–196, 99–305) [55],[56], two ICF patient fibroblast samples [57], and four IBMPFD patient samples (two fibroblast and two lymphoblast) (JH-FIB, MJ-FIB, 307/98, and RS-LCL) [58] were used. Control (256.1 LCL), ICF (10759 ICF LCL), and FSHD (B8-1) lymphoblast cells were grown in RPMI-1640 supplemented with 10% FBS, penicillin/streptomycin, and 2 mM L-Glutamine (Invitrogen-Gibco, CA). Human ES cells H1 and H9 were grown as described [59]. Mouse somatic cell hybrids containing chromosome 4, 10, 13, 14, 15 or 21 (GM11687, 11688, 11689, 10479, 11715, 08854, respectively, from Coriell Cell Repositories, Camden, NJ) were grown in DMEM/F-12 (1∶1) medium with the same supplements as the fibroblasts. Chromosomes 13, 14, 15, and 21 are known to contain D4Z4-like repeat sequences [60]. The NIGMS Human/Rodent Somatic Cell Hybrid Mapping Panel #2, version 3 was from Coriell Cell Repositories, in which chromosome 1, 16, 17, 20, and 21 hybrids are from mice while the others are from Chinese hamsters.

### Antibodies

Antigen affinity-purified rabbit polyclonal antibodies specific for Rad21, hSMC1, hCAP-G, and the pre-immune IgG control were published previously [53],[61]. Antibodies against H3K4me2, H3K4me3, H3K9me3, H3K27me3, H3 Ac, H4 Ac, HP1γ, SUV39H1, and G9a (Upstate Biotech, MA), against H3K9me3 (Abcam, Cambridge, MA) and against HP1α (Novus Biologicals, CO) were used. Antibody against 5-methylcytidine was from Eurogentec North America (San Diego, CA).

### ChIP analysis

The ChIP analysis was performed as recommended by the Upstate ChIP assay kit. Briefly, we crosslinked the cells with 1% formaldehyde and used 1×10^6^ cells for one histone ChIP and 3×10^6^ cells for the other ChIP assays. Protein A beads were preincubated with 1 mg/ml BSA and 0.2 mg/ml ssDNA for 20 min at 4°C. Typically, 4–8 µg of affinity-purified IgG was used per assay. The mixtures of antibody and nuclear extracts pre-cleared with protein A beads were incubated at 4°C overnight followed by precipitation with protein A beads. After washing, immunoprecipitated materials were eluted with 0.1 M NaHCO_3_ and 1% SDS, and crosslinks were reversed at 65°C for 4–6 hrs. Primer sequences are listed in Table S2. PCR primers specific for chromosome 1 α-satellite (α-sat) and satellite 2 (sat2), chromosome 4 α-satellite (α-sat), DXZ4, RS447, and NBL2 sequences were used [6],[12],[62]. In addition, a PCR primer pair specific for the c-Myc region was used as a control for G9a depletion as previously described [28]. The primers for rDNA are located in the intergenic region. All of the end-point PCR experiments were repeated at least three times. The endpoint gel quantitation of the ChIP-PCR products was carried out using the Gel-Doc Imager and Quantity One software (Bio-Rad). Real-time Q-PCR primers were designed using Lasergene software. Q-PCR was performed using the iCycler iQ Real-time PCR detection system (Bio-Rad) with iQ SYBR Green Supermix (Bio-Rad). The ChIP PCR signal was normalized by the subtraction of the preimmune IgG ChIP PCR signal, which was further divided by input genomic PCR (for normalization of different D4Z4 repeat numbers in different cells) minus PCR with no template. Results were an average of three PCR reactions, and the arbitrary value of 1.0 was assigned to the normal control sample. Double-ChIP analysis was performed according to the published protocol [63].

### 5-Azacytidine (5-AzaC) treatment and methylcytidine ChIP (MeCIP) assay

The 5-AzaC treatment was performed as previously described [64]. Briefly, 50 µM of 5-AzaC was added to HeLa cells at 80% confluency and after 24 hr incubation, the cells were harvested for ChIP experiments. The MeCIP assay was performed according to the published protocol [65]. After the cell samples were harvested and sonicated, they were treated with proteinase K overnight and the DNA from these samples was purified by the QIAquick gel purification kit (QIAGEN). Four µg of the purified DNA was used per MeCIP assay. The DNA was denatured at 95°C for 10 min and incubated with 4 µl antibody against 5-methylcytidine in 500 µl IP buffer (10 mM sodium phosphate, pH 7.0, 140 mM NaCl, 0.05% Triton X-100) at 4°C for 2 hrs. The DNA: antibody mixtures were further incubated with protein A beads at 4°C for an additional 2 hrs. The beads were washed with 700 µl IP buffer three times and treated with proteinase K at 50°C for 3 hrs. Finally, the DNA was recovered using the gel purification kit and analyzed by PCR.

### siRNA transfection

HeLa cells were transfected three times 24 hours apart with siRNAs at a final concentration of 10 nM using HiPerFect Transfection Reagent per manufacturer's instructions (Qiagen). The target sequences for SUV39H1 and G9a were previously described [66],[67]. Other siRNA target sequences include hSMC1 (5′-CACCATCACACTTTAATTCCA-3′), HP1γ (5′-CTAAGTTAAATGAACATTTAA-3′), Scc2 (5′-CTAGCTGACTCTGACAATAAA-3′), and negative control (5′-AATTCTCCGAACGTGTCACGT-3′). Cells were used for ChIP and western blot analyses at 48 hours after the third transfection.

### Coimmunoprecipitation (co-IP)–western analysis

HeLa nuclear extracts were used for co-IP using antibody specific for Scc2 or cohesin (Rad21) as previously described [53],[61]. Briefly, precipitated materials were washed four times with a buffer containing 0.1 M KCl, then eluted with 1.0 M KCl (“wash”) and finally eluted with 2.0 M guanidine-HCl (“eluate”). Proteins in the wash and eluate fractions were precipitated by trichloroacetic acid (TCA) and analyzed by SDSPAGE and western blotting using antibody specific for HP1γ.

## Supporting Information

Figure S1 H3K9me3 ChIP analysis of different repeat sequences in normal and FSHD patient cells. PCR primers specific for chromosome 1 α-satellite (α-sat) and satellite 2 (sat2) and chromosome 4 α-satellite (α-sat) sequences were used. In addition, DXZ4, RS447, and NBL2-specific primers were used. Although sequences are unrelated, DXZ4 (on Xq23) and RS447 (primarily on 4q16.1) are members of the macrosatellite repeat family similar to D4Z4. NBL2 is in the acrocentric chromosomes and is known to be DNA-hypomethylated in ICF syndrome patient cells (see Figure 4B). The PCR primer sequences are listed in the Supporting Table S2. Results of the endpoint PCR using 4qHox primers and realtime PCR using Q-PCR primers for (A) myoblasts (normal (N27) and 4qF (GM17940)), (B) fibroblasts (normal (KI-I), PF (KII-I), and 4qF (RD217)), and (C) lymphoblasts (normal (256) and 4qF (B8-1)) are shown.
(1.13 MB TIF)

Figure S2 Cohesin and HP1γ binding to different repeat sequences. Rad21 and HP1γ ChIP analysis of three repeat sequences (α-sat and sat2 on chromosome 1 and DXZ4) in normal and FSHD myoblasts, fibroblasts, and lymphoblasts as indicated. Endpoint PCR using 4qHox primers and realtime PCR analysis using Q-PCR primers are shown.
(0.70 MB TIF)

Table S1 The number of input and ChIP DNA PCR clones with 4q- or 10q-specific nucleotide polymorphisms. Input and ChIP DNA amplified by Q-PCR primer pairs was cloned and sequenced to identify the chromosome of origin based on SNPs that allow us to distinguish 4q- and 10q-derived D4Z4 sequences.
(0.05 MB DOC)

Table S2 List of PCR primers used.
(0.06 MB DOC)
