# Supplementary figures and images for: Specific Loss of Histone H3 Lysine 9 Trimethylation and HP1γ/Cohesin Binding at D4Z4 Repeats Is Associated with Facioscapulohumeral Dystrophy (FSHD)
Source: PLoS Genet. 2009 Jul 10;5(7):e1000559. doi: 10.1371/journal.pgen.1000559 (PMC2700282; doi:10.1371/journal.pgen.1000559)

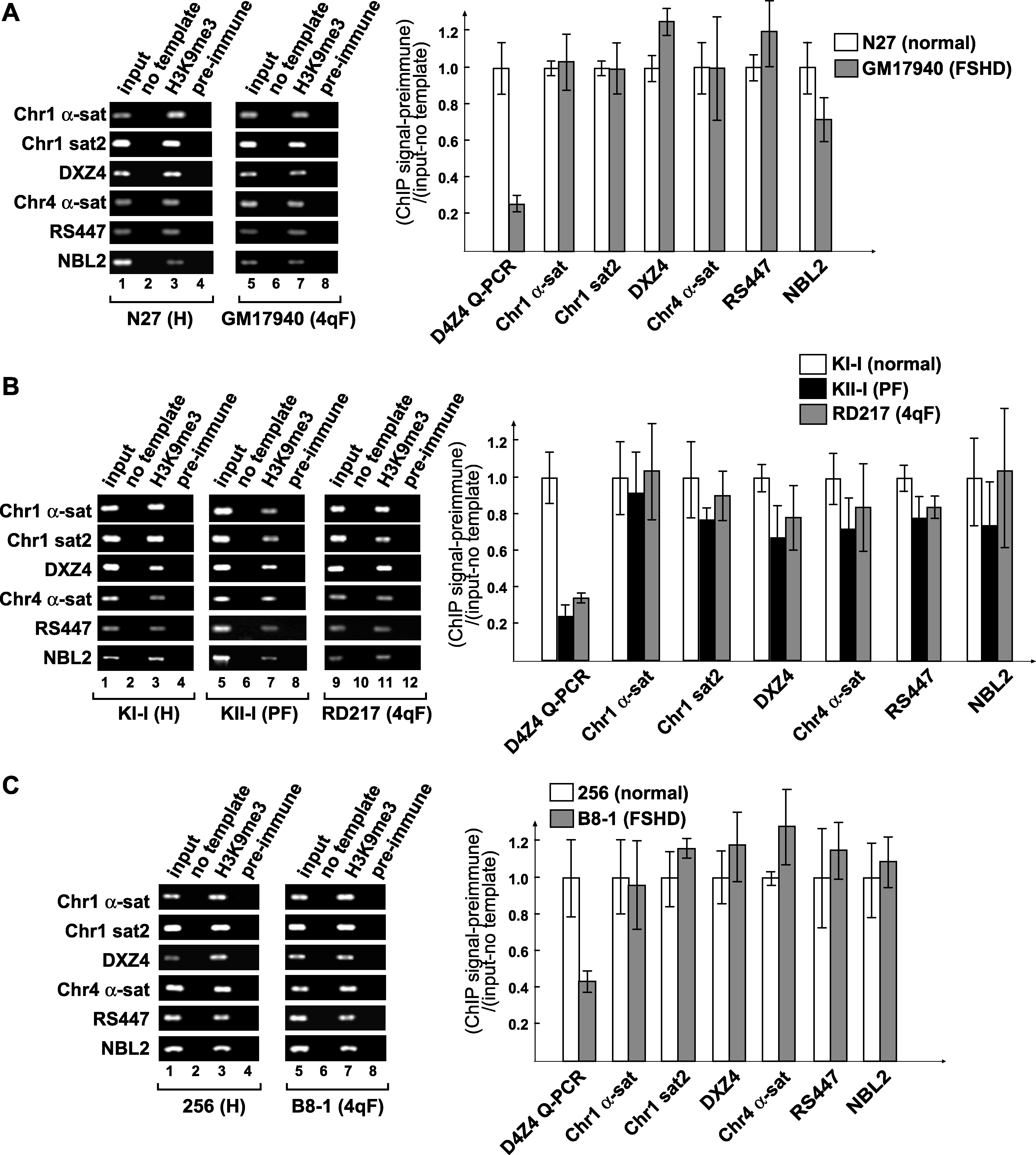

Supplement: Figure S1 — H3K9me3 ChIP analysis of different repeat sequences in normal and FSHD patient cells. PCR primers specific for chromosome 1 α-satellite (α-sat) and satellite 2 (sat2) and chromosome 4 α-satellite (α-sat) sequences were used. In addition, DXZ4, RS447, and NBL2-specific primers were used. Although sequences are unrelated, DXZ4 (on Xq23) and RS447 (primarily on 4q16.1) are members of the macrosatellite repeat family similar to D4Z4. NBL2 is in the acrocentric chromosomes and is known to be DNA-hypomethylated in ICF syndrome patient cells (see Figure 4B). The PCR primer sequences are listed in the Supporting Table S2. Results of the endpoint PCR using 4qHox primers and realtime PCR using Q-PCR primers for (A) myoblasts (normal (N27) and 4qF (GM17940)), (B) fibroblasts (normal (KI-I), PF (KII-I), and 4qF (RD217)), and (C) lymphoblasts (normal (256) and 4qF (B8-1)) are shown. (1.13 MB TIF) [file pgen.1000559.s001.tif]

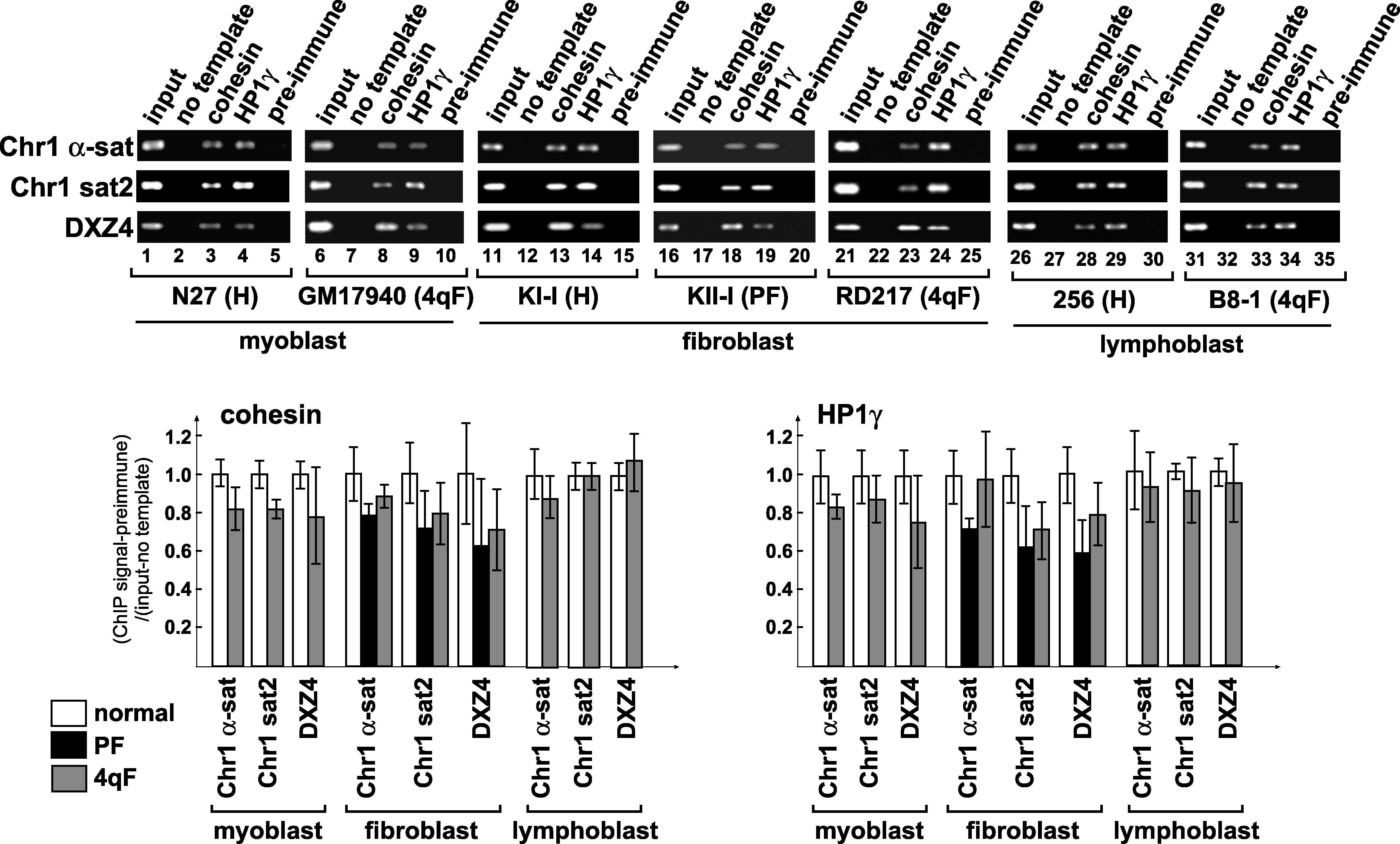

Supplement: Figure S2 — Cohesin and HP1γ binding to different repeat sequences. Rad21 and HP1γ ChIP analysis of three repeat sequences (α-sat and sat2 on chromosome 1 and DXZ4) in normal and FSHD myoblasts, fibroblasts, and lymphoblasts as indicated. Endpoint PCR using 4qHox primers and realtime PCR analysis using Q-PCR primers are shown. (0.70 MB TIF) [file pgen.1000559.s002.tif]
