# Supplementary material for: Specific Loss of Histone H3 Lysine 9 Trimethylation and HP1γ/Cohesin Binding at D4Z4 Repeats Is Associated with Facioscapulohumeral Dystrophy (FSHD)
Source: PLoS Genet. 2009 Jul 10;5(7):e1000559. doi: 10.1371/journal.pgen.1000559 (PMC2700282; doi:10.1371/journal.pgen.1000559)
Supplement: Table S1 — The number of input and ChIP DNA PCR clones with 4q- or 10q-specific nucleotide polymorphisms. Input and ChIP DNA amplified by Q-PCR primer pairs was cloned and sequenced to identify the chromosome of origin based on SNPs that allow us to distinguish 4q- and 10q-derived D4Z4 sequences. (0.05 MB DOC) [file pgen.1000559.s003.doc]

**Table S1.** The number of input and ChIP DNA PCR clones with 4q- or 10q-specific nucleotide polymorphisms.

| **Fibroblasts** | 4q D4Z4 | 10q D4Z4 |
| --- | --- | --- |
| **Normal (KI-I)** | | |
| input | 5 | 2 |
| H3K4me2 | 4 | 5 |
| H3K9me3 | 3 | 3 |
| HP1γ | 2 | 5 |
| cohesin | 6 | 2 |
| **Phenotypic FSHD (KII-I)** | | |
| input | 5 | 2 |
| H3K4me2 | 4 | 3 |
| H3K9me3 | 3 | 2 |
| **4q-linked FSHD (RD217)** | | |
| input | 1 | 6 |
| H3K4me2 | 7 | 3 |
| H3K9me3 | 4 | 2 |

| **Normal lymphoblasts** | 4q D4Z4 | 10q D4Z4 |
| --- | --- | --- |
| input | 3 | 3 |
| H3K9me3 | 4 | 3 |

| **Normal myoblasts** | 4q D4Z4 | 10q D4Z4 |
| --- | --- | --- |
| input | 2 | 5 |
| H3K4me2 | 4 | 10 |
| H3K9me3 | 4 | 10 |
| HP1γ | 3 | 11 |
| cohesin | 2 | 12 |

| **HeLa** | 4q D4Z4 | 10q D4Z4 |
| --- | --- | --- |
| cohesin | 4 | 3 |
| HP1γ | 3 | 5 |
