# Supplementary material for: Specific Loss of Histone H3 Lysine 9 Trimethylation and HP1γ/Cohesin Binding at D4Z4 Repeats Is Associated with Facioscapulohumeral Dystrophy (FSHD)
Source: PLoS Genet. 2009 Jul 10;5(7):e1000559. doi: 10.1371/journal.pgen.1000559 (PMC2700282; doi:10.1371/journal.pgen.1000559)
Supplement: Table S2 — List of PCR primers used. (0.06 MB DOC) [file pgen.1000559.s004.doc]

**Table S2.** List of PCR primers used.

| 4qHox forward | 5’CGAGGACGGCGACGGAGAC3’ |
| --- | --- |
| 4qHox reverse | 5’ACCCTGTCCCGGGTGCCTG3’ |
| Q-PCR forward | 5’CCGCGTCCGTCCGTGAAA3’ |
| Q-PCR reverse | 5’TCCGTCGCCGTCCTCGTC3’ |
| rDNA 445 | 5’CATAAGTGTGTGTTCCCGTGAGG3’ |
| rDNA 446 | 5’CCTAGCCCAGTAGCAATACAGTGC3’ |
| rDNA 347 | 5’TGAAACCCCGTCTCTACTCAC3’ |
| rDNA 348 | 5’CGAAACATCAATCATGATAATAAC3’ |
| Mouse β-minor globin forward | 5’TGCGAGGATAAGAACAGACACTAC3’ |
| Mouse β-minor globin reverse | 5’ACAGACTCAGAAGCAAACGTAAGA3’ |
| Chinese hamster rDNA forward | 5’GCAGAAGCTGCCAGGATAAC3’ |
| Chinese hamster rDNA reverse | 5’AGGGGTGGTGTCTTTGACAG3’ |
| Sat2 from Chr1 forward | 5’CATCGAATGGAAATGAAAGGAGTC3’ |
| Sat2 from Chr1 reverse | 5’ACCATTGGATGATTGCAGTCAA3’ |
| Satα from Chr1 forward | 5’TCATTCCCACAAACTGCGTTG3’ |
| Satα from Chr1 reverse | 5’TCCAACGAAGGCCACAAGA3’ |
| Satα from Chr4 forward | 5’CTGCACTACCTGAAGAGGAC3’ |
| Satα from Chr4 reverse | 5’GATGGTTCAACACTCTTACA3’ |
| NBL2 forward | 5’TGTTCGTCTTTGCAGTTGTCCT3’ |
| NBL2 reverse | 5’TCCACTCCTGACAGATAGGCTG3’ |
| DXZ4 forward | 5’GCCTACGTCACGCAGGAAG3’ |
| DXZ4 reverse | 5’ TATGTTTGGGCAGGAAGATCG3’ |
| RS447 forward | 5’TGGGAAATACCTGCTACGTG3’ |
| RS447 reverse | 5’GTGACGATGACACGTTTGAG3’ |
| Chr10 Alu forward | 5’GATTCTCAACAGCAGAATTCCATGCC3’ |
| Chr10 Alu reverse | 5’CATGTTTGAGAATGTCTACTTCTTAG3’ |
| Chr19 Alu forward | 5’CCACGTGTTTATCTGTAAGGTG3’ |
| Chr19 Alu reverse | 5’GTTAGGAGCTAGAAGGAGCCTG3’ |
